# Relationship between Depressive Symptoms and Cardiovascular Disease Risk Factors in African American Individuals

**DOI:** 10.1155/2011/836542

**Published:** 2011-08-04

**Authors:** Ali A. Weinstein, Preetha Abraham, Guoqing Diao, Stacey A. Zeno, Patricia A. Deuster

**Affiliations:** ^1^Center for the Study of Chronic Illness and Disability, George Mason University, 4400 University Drive, MSN 5B7, Fairfax, VA 22030, USA; ^2^Department of Military and Emergency Medicine, Uniformed Services University of the Health Sciences, Bethesda, MD 20814, USA; ^3^Department of Statistics, George Mason University, Fairfax, VA 22030, USA

## Abstract

*Objective*. To examine the relationship between depressive symptoms and cardiovascular disease (CVD) risk factors in a group of African American individuals. *Design*. A nonrandom sample of 253 (age 43.7 ± 11.6 years; 37% male) African American individuals was recruited by advertisements. Data were obtained by validated questionnaires, anthropometric, blood pressure, and blood sample measurements. *Results*. Regression analyses were performed to assess the relationship between depressive symptoms and CVD risk factors controlling for socioeconomic status indicators. These analyses demonstrated that those with higher levels of depressive symptoms had larger waist-to-hip ratios, higher percent body fat, higher triglycerides, and were more likely to be smokers. *Conclusions*. It has been well documented that higher levels of depressive symptoms are associated with higher CVD risk. However, this evidence is derived primarily from samples of predominantly Caucasian individuals. The present investigation demonstrates that depressive symptoms are related to CVD risk factors in African American individuals.

## 1. Introduction

African Americans are at a greater risk for cardiovascular disease (CVD) and stroke [1], having higher rates of obesity and lower exercise capacity [2] than their Caucasian counterparts; they also suffer premature mortality because of CVD [1]. To eliminate these disparities, it is essential to understand why these differences may occur. Studies have shown that socioeconomic status underlies a substantial portion, but not all, of the higher rate of CVD and CVD consequences in African American individuals [3]. However, other factors must be examined to try to explain this disproportionate burden of CVD.

The factors that may help to explain potential health disparities are not well understood. African Americans may be at an increased risk of CVD (and increased consequences once CVD has been developed) due to increased exposure to stress (i.e., racial discrimination) [4]. This increased exposure to stress may influence mental health outcomes, such as depression [5]. Depression has been linked to several adverse cardiovascular outcomes and may be important to consider when investigating potential disparities in CVD. In addition, evidence exists that the prevalence and severity of depression may differ in African Americans when compared to other racial groups [6].

Depression is associated with several physiological abnormalities that could contribute to adverse cardiac outcomes [7, 8]. Many individuals with depression have high sympathetic tone, hypercholesterolemia, elevated catecholamine levels, abnormal platelet activation, increased inflammatory markers, and endothelial dysfunction compared to persons without depression [9]. Importantly, these physiological abnormalities are present in depressed individuals who do not have cardiac disease [10].

Despite the evidence for physiological mechanisms, it is equally likely that behavioral mechanisms partially or wholly explain the association between depression and CVD [7, 8]. Compared with individuals who are not depressed, individuals with depression are significantly less likely to adhere to prescribed medications, follow healthy lifestyle recommendations, and practice self-management techniques. However, previous research (both physiological and behavioral) conducted in this area was focused primarily on Caucasian individuals. Therefore, the present investigation examined the relationship between depressive symptoms and traditional CVD risk factors in a group of African American individuals.

## 2. Methods

### 2.1. Participants

African American males and females between the ages of 18–60 were recruited from the Washington DC metropolitan area community. Participants were excluded from participation if they were pregnant. These participants were recruited using advertisements in local newspapers, flyers posted in the community, and website-based recruiting sites (such as Craig's List). Participants were completely informed as to the nature of the study and signed an informed consent document if they wished to participate. The research was reviewed and approved by the Institutional Review Board of the Uniformed Services University of the Health Sciences. A total sample of 253 participants was recruited for this investigation.

### 2.2. Procedures

After arriving at the testing location, participants completed a medical history and demographic information questionnaire (ethnicity, education, income), as well as various other questionnaires (discussed in detail below). In addition, participant assessments consisted of anthropometric measures (weight, height, waist and hip circumferences, and percent body fat), blood pressure, blood draws for fasting lipid profiles (total cholesterol, triglycerides, high-density cholesterol (HDL), and calculated low-density lipoprotein (LDL)), and blood glucose level.

### 2.3. Assessments

#### 2.3.1. Blood Parameters

Fasting blood was collected between 0800 and 0900 hr. The participants were asked not to eat after midnight the night before the session. A trained technician collected samples in anticoagulant tubes for the lipid profiles. Plasma was extracted and stored in an −80°C freezer. Lipid profiles were determined at the National Institutes of Health (NIH), Department of Laboratory Medicine by using a LX-20 analyzer (Beckman, San Diego, Calif). LDL was calculated by the Friedewald equation [11]. Blood glucose concentration was measured with the OneTouch Ultra by Life Scan, Inc (Milpitas, Calif).

#### 2.3.2. Blood Pressure

One-hour resting blood pressure and heart rate were taken in the supine position with the Criticare Systems automatic blood pressure machine (model 506N3; Waukesha, Wis).

#### 2.3.3. Anthropometric Measures

Body-mass index (BMI) was calculated from the anthropometric measures as weight in kilograms as divided by height in squared in meters. Weight and height were taken with participants in light clothing using the Cardinal Detecto Physician's Scale. Percent body fat was calculated from bioelectric impedance by using the National Health and Nutrition Examination Survey formula [12].

#### 2.3.4. Questionnaires

**Center for Epidemiological Studies Depression Scale (CES-D)**

The CES-D is a self-report scale with 20 items, each of which is rated on a 4-point scale, with a minimum score of zero and a maximum score of 60. Individuals are asked to report the frequency of how they felt in the previous week on parameters such as crying spells, loneliness, self-esteem, and sleep [13]. Scores of 16 or greater on the CES-D are traditionally interpreted as suggestive of clinically significant depression [14]. Among community samples, internal consistency estimates range from 0.8 to 0.9; test-retest reliability, ranging from 25 weeks to one year, is reported to be between 0.4 to 0.7 [13, 15].

**Pittsburgh Sleep Quality Index (PSQI)**

The PSQI was developed to measure sleep quality during the previous month and to discriminate between good and poor sleepers [16]. The PSQI is composed of 19 questions. The self-administered scale contains 15 multiple-choice items that inquire about frequency of sleep disturbances and subjective sleep quality and four write-in items that inquire about typical bedtime, wake-up time, sleep latency, and sleep duration. The PSQI generates seven component scores that correspond to various sleep disturbance domains. Each component score ranges from 0 (no difficulty) to 3 (severe difficulty). The component scores are summed to produce a global score (range of 0–21). A PSQI global score >5 is considered to be suggestive of significant sleep problems. Internal consistency is 0.83 (Cronbach's *α*) for the global score [16]. Test-retest reliability (average interval of 28 days) is 0.85 for the global score [17].

**Perceived Stress Scale (PSS)**

The PSS is a 4-item self-report questionnaire that measures persons' evaluation of the stressfulness of the situations in the past week of their lives [18]. Each question on the PSS is scored on a five-point scale from 0 (never) to 4 (very often), and scores can range from 0 to 16, with higher scores indicating greater stress. The reliability of the PSS has been found to be good (Cronbach's *α* = 0.84–0.86) [18].

### 2.4. Statistical Analyses

We performed descriptive analysis for all variables of interest. To compare the demographic, physiological, and psychological characteristics of those at or above the depressive symptom threshold to those below the threshold, we conducted independent group *t*-tests for continuous data and chi-square analyses for categorical variables. In addition, we performed regression analysis to assess the effects of depressive symptoms on cardiovascular risk factors while controlling for potential confounding demographic variables that differed between the groups (income, employment, and education). Within the regression analyses, these potential confounding variables were treated as binary variables, and depressive symptom (CES-D score) was treated as a continuous variable. For continuous dependent variables, we first applied normal score transformation and then fit simple linear regression models. Normal score transformation was utilized in order to approximate normality. For the binary dependant variable of smoking, we fit a logistic regression model.

## 3. Results

Of the 253 participants, 96 (34%) had depression symptoms at or above the threshold (CES-D score ≥ 16) as compared with 157 (66%) who were below the threshold. Individuals at or above the depression symptom threshold were less likely to be currently employed, had a lower annual family income, and had reached a lower educational level compared to those individuals below the depression symptom threshold (Table 1).

Overall, the majority of the sample was obese (defined as BMI > 30). Individuals at or above the depression threshold had higher levels of triglycerides (*P* = 0.03) than individuals below the threshold. The percentage of participants that identified themselves as smokers was significantly higher in the individuals at or above the depression threshold compared to those below the threshold level (*P* < 0.01). No other CVD risk factors (blood parameters, blood pressure, and body composition) differed significantly between the groups. Individuals with depression scores at or above the threshold also had higher scores on the PSQI (*P* < 0.001) and the PSS (*P* < 0.001) (Table 2).

The individuals at or above the depression symptom threshold differed from those below the depression threshold on the demographic variables of employment, income, and education level (key parameters of socioeconomic status). Therefore, we conducted regression analyses controlling for these variables to determine if there were consistent relationships between depressive symptoms and traditional risk factors. These analyses demonstrated that those with higher levels of depressive symptoms had larger waist-to-hip ratios (*P* = 0.02), higher percent body fat (*P* = 0.03), and higher triglycerides (*P* = 0.04) and were more likely to be smokers (*P* < 0.01) (Table 3).

## 4. Discussion

The purpose of this study was to compare various CVD risk factors between individuals who have depressive symptoms at or above threshold with those who do not among a group of African Americans. The percentage of African American participants above the depression threshold level was 34%, which is substantially higher than what has been reported in a sample where the majority of the individuals were Caucasian (10%) [19]. This substantial proportion of individuals with depressive symptoms above the threshold highlights the importance of routinely assessing for depression in African Americans: individuals with depression have higher mortality rates and are less likely to engage in healthy lifestyle behaviors than their nondepressed counterparts [20, 21]. 

A positive relationship between depression and CVD risk has been well documented in Caucasian samples [22]. However, this association has not been well investigated in African American samples. The present investigation demonstrates that depressive symptoms are related to traditional CVD risk factors (specifically, waist-to-hip ratio, percent body fat, triglycerides, and smoking status).

We recognize several limitations to our study design and methodology. The analyses presented are exploratory in nature. Therefore, the current results should be replicated in studies that establish these hypotheses *a priori*, as the current study did not. Also, causal inference and temporality cannot be implied because our analyses were based on cross-sectional data. For example, it has been well established that the relationship between depression and obesity is bidirectional, and therefore we cannot state that depressive symptoms “caused or preceded” obesity nor obesity “caused or preceded” depressive symptoms. Additionally, our sample was conveniently selected and restricted to African Americans, which limits the generalizability of our results, and depressive symptoms were evaluated through a self-rating scale rather than the gold standard structured clinical interview, which may limit the validity of the assessment of depressive symptoms. We investigated a selective number of covariates in our statistical analyses, and therefore it is possible that other variables that we have not assessed and residual confounding in assessed variables could confound our results. In addition, we do not have a comparison group of Caucasian individuals. Nonetheless, the findings are striking and provide a basis to further explore the role of depressive symptoms in CVD as well as provide insights into questions to ask when working with African Americans at risk for developing CVD.

In conclusion, CVD is the leading cause of death in the United States, and disproportionate rates are seen in African American individuals [1]. It is important to determine the causes for this discrepancy. One potential explanation for this disparity was investigated in the current study: the relationship between depressive symptoms and CVD risk factors. In this sample of African American individuals, we found a high number of depressive symptoms and these symptoms were related to traditional CVD risk factors. Future research will need to examine these relationships in a longitudinal manner to determine if the presence of depressive symptoms is a precursor to the development of CVD disease in African American individuals.

## Figures and Tables

**Table 1:** Participant characteristics (categorical variables).

		Total	CES-D ≥ 16	CES-D < 16	Pearson Chi-Square	*df*	*P* value
		*N* = 253	*N* = 96	*N* = 157			
Gender	Male (%)	94 (37%)	32 (34%)	62 (39%)	1.00	1	0.33

Marital status	Married (%)	43 (17%)	14 (15%)	29 (19%)	6.12	5	0.41
	Living with significant other (%)	5 (2%)	2 (2%)	3 (2%)			
	Separated/divorced (%)	53 (21%)	21 (22%)	32 (21%)			
	Widowed	8 (3%)	3 (3%)	5 (3%)			
	Single/never married (%)	141 (56%)	56 (58%)	85 (54%)			
	Would rather not say (%)	3 (1%)	1 (1%)	2 (1%)			

Highest level of education	Grammar school (%)	5 (2%)	3 (3%)	2 (1%)	23.5	7	<0.01
	High school diploma (%)	59 (23%)	30 (31%)	29 (19%)			
	Vocational/technical school (%)	22 (9%)	13 (14%)	9 (6%)			
	Some college (%)	72 (28%)	27 (28%)	45 (29%)			
	Bachelor's degree (%)	37 (15%)	10 (11%)	27 (17%)			
	Some graduate school (%)	21 (8%)	6 (6%)	15 (10%)			
	Graduate degree (%)	32 (13%)	4 (4%)	28 (18%)			
	Would rather not say (%)	4 (2%)	3 (3%)	1 (1%)			

Annual family income	<$19,000 (%)	77 (31%)	39 (41%)	38 (24%)	21.9	5	<0.01
	$20,000–$49,999 (%)	69 (27%)	25 (26%)	44 (28%)			
	$50,000–$79,999 (%)	32 (13%)	7 (8%)	25 (16%)			
	$80,000–$99,999 (%)	15 (6%)	1 (1%)	14 (9%)			
	>$100,000 (%)	24 (10%)	6 (6%)	18 (12%)			
	Would rather not say (%)	33 (13%)	17 (18%)	16 (11%)			

Currently working?	Yes (%)	142 (56%)	43 (45%)	99 (63%)	8.20	1	<0.01

Current smoker?	Yes (%)	93 (36%)	46 (48%)	47 (30%)	8.47	1	<0.01

**Table 2:** Participant characteristics (continuous variables).

	Total	CES-D ≥ 16	CES-D < 16	*t*-Test	*df*	*P* value
	*N* = 253	*N* = 96	*N* = 157			
Age (years) ± SD	43.7 ± 11.6	44.9 ± 11.0	42.8 ± 11.8	1.33	251	0.19
Body-mass index (kg/m^2^) ± SD	30.3 ± 8.5	30.6 ± 8.3	30.2 ± 8.7	0.45	251	0.66
Waist to hip ratio ± SD	0.85 ± 0.1	0.87 ± 0.1	0.84 ± 0.1	1.79	250	0.08
Body-fat% ± SD	34.5 ±10.6	35.5 ± 10.8	34.0 ± 10.5	1.11	249	0.27
Heart rate (beats/min) ± SD	70.5 ± 12.0	71.7 ± 12.1	70.0 ± 11.7	1.08	251	0.28
Systolic BP (mmHg) ± SD	132.4 ± 16.7	132.1 ± 17.6	132.3 ± 16.2	0.08	251	0.94
Diastolic BP (mmHg) ± SD	82.5 ± 12.8	82.3 ± 12.4	82.5 ± 12.9	0.10	251	0.92
Cholesterol (mg/dL) ± SD	156.9 ± 37.4	160.5 ± 38.2	154.8 ± 37.0	1.17	251	0.25
HDL (mg/dL) ± SD	49.6 ± 15.3	51.5 ± 16.8	48.5 ± 14.3	1.49	251	0.14
LDL (mg/dL) ± SD	85.1 ± 32.6	85.4 ± 31.2	85.9 ± 31.4	0.51	251	0.61
Triglycerides (mg/dL) ± SD	111.2 ± 85.6	125.9 ± 114	101.7 ± 61.0	2.20	251	0.03
Glucose (mg/dL) ± SD	110.1 ± 29.4	110.9 ± 23.1	109.6 ± 32.8	0.34	250	0.74
PPAQ daily activity (METS) ± SD	74.9 ± 21.9	75.0 ± 24.1	74.5 ± 19.9	0.17	239	0.87
Global PSQI score ± SD	7.3 ± 3.9	9.2 ± 3.9	6.2 ± 3.5	6.43	251	<0.01
PSS ± SD	5.0 ± 3.3	7.4 ± 2.9	3.5 ± 2.5	11.3	251	<0.01

SD: standard deviation; BP: blood pressure; HDL: high-density lipoprotein; LDL: low-density lipoprotein; PPAQ: Paffenbarger physical activity questionnaire; PSQI: Pittsburgh sleep quality index; PSS: perceived stress scale.

**Table 3:** Relationship between depressive symptoms and traditional CVD risk factors while controlling for socioeconomic status.

	*R* ^2^ *H*_0_	*R* ^2^ *H*_1_	*β* (s.e.)	Wald test statistic	*df*	*P*-value
Body-mass index (kg/m^2^)	0.01	0.02	0.01 (0.007)	2.17	1	0.14
Waist-to-hip ratio	0.08	0.10	0.02 (0.008)	5.24	1	0.02
Body-fat%	0.01	0.03	0.02 (0.007)	4.95	1	0.03
Heart rate (beats/min)	0.01	0.01	0.01 (0.008)	2.15	1	0.14
Systolic BP (mmHg)	0.06	0.06	0.01 (0.007)	0.94	1	0.33
Diastolic BP (mmHg)	0.04	0.04	0.01 (0.007)	0.02	1	0.89
Cholesterol (mg/dL)	0.02	0.03	0.01 (0.008)	1.99	1	0.16
HDL (mg/dL)	0.02	0.03	0.01 (0.007)	2.16	1	0.14
LDL (mg/dL)	0.03	0.03	0.01 (0.008)	0.03	1	0.86
Triglycerides (mg/dL)	0.02	0.04	0.02 (0.008)	4.12	1	0.04
Glucose (mg/dL)	0.03	0.04	0.01 (0.008)	0.97	1	0.25
Current smoker	N/A	N/A	0.06 (0.018)	10.92	1	<0.01

*R* ^2^ *H*_0_: Regression model includes income, education, and employment status; *R* ^2^ *H*_1_: Regression model includes income, education, employment status, and score on the Center for Epidemiological Studies Depression Scale; N/A: Not applicable since current smoker is a binary variable, and therefore a logistic regression model was fitted; Traditional CVD risk factors were modeled separately; BP: blood pressure; HDL: high-density lipoprotein; LDL: low-density lipoprotein.
